# Rheumatoid vasculitis: going, going, but not yet gone

**DOI:** 10.1186/s13075-015-0635-0

**Published:** 2015-05-08

**Authors:** David S Pisetsky

**Affiliations:** Medical Research Service, Durham VA Medical Center, Duke University Medical Center, Durham, NC 27710 USA

Recently, one of our fellows hurried into the small room where I hang out when I attend in the clinic. The room is painted a bland yellow and is furnished with only an examining table and a desk with a computer that is the portal to our electronic medical record. The fellow looked worried and wanted advice on a physical finding, its strangeness clearly alarming her.

‘The patient has black spots next to his nails,’ she said excitedly. ‘What should I do?’

‘Does the patient have rheumatoid arthritis,’ I asked, looking away from the computer where I was fiddling with a Disease Activity Score calculator, testing how various values for the sedimentation rate can influence proximity to the target.

‘Yes’, she said.

‘Is anything else going on? Fever? Malaise? Neuropathy?’

‘No’, she said.

‘Probably it’s nothing to worry about then. Those are nail fold infarcts’, I said calmly, amused that the fellow had to ask me at all. ‘It’s sometimes called bland vasculitis’.

The word vasculitis startled the fellow and ratcheted up the concern in her face. ‘Vasculitis?’ she said, her eyes widening. ‘Should I admit the man to the hospital? Should I start steroids?’ The questions came in rapid succession.

‘No’, I said. The particular fellow regularly looks at me with an expression of skepticism if not puzzlement, seemingly surprised that the term vasculitis had not stirred a greater response. ‘Let’s go see him’.

The fellow and I then went to see the patient. The man looked about 60 years old but had the frail appearance of someone who had suffered rheumatoid arthritis (RA) for many years. For whatever reason, he had missed out on modern aggressive therapy, the target never hit or perhaps even aimed at. His fingers were angulated and there were nodules on top of his knuckles. At the juncture of a few of his nails, there were black spots that were slightly textured but not tender. These were definitely the nail fold infarcts of RA.

With the diagnosis confirmed, I chatted briefly with the patient, performed a joint examination (calculating a low Disease Activity Score despite the deformities) and asked the fellow to come back to my room so that we could review rheumatoid vasculitis. I wanted to explain to her the various forms of vessel involvement in RA: leukocytoclastic; polyarteritis-like with mononeuritis multiplex, digital gangrene and skin ulcers; and nail fold infracts.

To show her the findings, I Googled rheumatoid vasculitis and pulled up images whose appearance startled me. There were black fingers that looked mummified, large gaping wounds covered with blood and pus, and a lower extremity with purpuric lesions that looked like the aftermath of a shotgun blast. Compared with those menacing lesions, the pictures of the nail fold infarcts looked benign.

My fellow, like many others today, has cared for tens if not hundreds of patients with RA. Would any believe that this disease once regularly produced the devastation of vasculitis or, for that matter, the dreaded complications of Felty’s syndrome, amyloidosis or scleritis? Scleritis could seriously jeopardize the eye, thinning the sclera so that the vitreous appeared an ominous blue–black and about to burst out.

As scholars of disease have noted, RA today appears to be a less severe disease than it was when I started my career in the 1970s. Then, patients routinely ‘went up in smoke’ and rapidly became crippled, wasting away in a state we called ‘burnt out’. Life expectancy with advanced RA could approach that of a malignancy but now, in some studies, patients with RA do as well as the general population. While the effectiveness of modern therapy can definitely improve outcomes in RA, the explanation for a moderate disease course is not fully known although smoking is down and the microbiome may have shifted, gaining or losing some species members.

Rightly, remission in RA – not just improvement – is now the target of therapy and current studies are addressing not only whether remission is possible – unquestionably, it is – but whether continued medication is necessary to maintain that state. These studies (for example, the PRIZE, PRESERVE and AVERT trials among others) concern patients with disease of varying duration and activity and involve initial treatment with a combination of methotrexate and a biological (etanercept, a tumor necrosis factor (TNF) blocker, or abatacept, a T-cell costimulatory blocker). The results of these studies are impressive: the rates of remission or low disease activity with the combination are high; the dose of the biological can be reduced or eliminated and low disease activity often maintained; and in some patients treated with a combination initially, remission can be maintained following eventual discontinuation of both agents. Even if the number of patients who go drug free is small, the results are nevertheless encouraging.

Few studies have addressed the need for sustained therapy with only classical small molecule disease modifying anti-rheumatic drugs such as methotrexate or sulfasalazine, but it is likely that some patients can similarly reduce or stop these drugs when remission occurs. Among those with sustained remission under whatever regimen, the arthritis may be more amenable to therapy – the window of opportunity opened more widely – because the immune system has not yet been permanently reconfigured (perhaps by epigenetic modification), a period of disease modifying anti-rheumatic drug treatment allowing restitution.

While there may be concern about flares at any time when the intensity of therapy in RA is lowered, data suggest that reinstitution of therapy can recapture a response. There is also the worry that, despite clinical remission, synovitis persists and grumbles, with the conditions that eventually lead to complications such as vasculitis not fully extinguished. Longer term follow-up is necessary to determine the fate of those patients whose medication has been reduced or eliminated.

Given these treatment results observed in practice as well as clinical trials, it may not be surprising that the frequency of RA vasculitis has been decreasing steadily. Interestingly, however, TNF blockers – a mainstay of current therapy contributing to improved outcomes – may themselves be associated with vasculitis. This complication, which can be manifested by leukocytoclastic vasculitis on skin biopsy, appears quite rare; it is, of course, always difficult to ascribe the development of a complication to a therapy rather than the course of disease. Nevertheless, the improvement of the vasculitis with cessation of the TNF blocker argues that such lesions can in fact be manifestations of drug toxicity.

Although the control of disease activity in RA is better today than in the past, vasculitis is still a problem for some patients and is likely to persist in health care systems and parts of the world where access to current therapy may be limited and disease progresses unchecked. Unfortunately, the improvements observed for RA do not seem to pertain to the vasculitis itself when it does occur. Even with our current armamentarium of biological and small molecule immunosuppressives, rheumatoid vasculitis of the severe necrotizing variety remains a dire condition that frequently resists therapy. Along with the occurrence of vasculitis that may occur with TNF blockers, these findings raise questions about the role of cytokines (and other treatment targets) in the vascular lesion in contrast to the arthritis.

For today’s rheumatologists, the landscape of many rheumatologic conditions – RA, ankylosing spondylitis, psoriatic arthritis – is vastly different from that just a generation ago. These changes are undoubtedly influenced by modern approaches to diagnosis and treatment, especially early aggressive therapy to treat to target with effective agents. I am glad I have been around to watch this transformation and, next time my fellow comes into my office to ask me about a toe that looks like a sausage, I will have my memory to tell her the meaning of the finding. I will, of course, have as a help that other great teacher of modern medicine – Google – to give her an image that she can remember even if, in regular practice, she never sees it again.

## Abbreviations

RA: Rheumatoid arthritis
TNF: Tumor necrosis factor
